# Ultra-processed foods and accelerated aging in adults: an emerging public health challenge for nutrition and functional health

**DOI:** 10.3389/fnut.2026.1807557

**Published:** 2026-04-21

**Authors:** Rosario Suárez, Manuel Celi, Karen Ontaneda, Dolores Jima-Gavilanes, Andri Matos

**Affiliations:** 1 Health Science Department, School of Medicine, Universidad Técnica Particular de Loja, Loja, Ecuador; 2 Universidad Espíritu Santo, Escuela de Medicina, Samborondón, Ecuador; 3 School of Allied Health, Eastwick College, Ramsey, NJ, United States

**Keywords:** biological aging, community health care, health care policies, healthy aging, ultra-processed foods

## Abstract

Ultra-processed foods (UPFs) intake has rapidly increased in global diets, posing a mounting threat to nutritional adequacy and functional health in adults. This narrative review summarizes recent evidence on the relationship between UPF consumption and functional decline, including sarcopenia and reduced handgrip strength, and potential biological mechanisms underlying the detrimental effects of high UPF consumption and aging processes. We examined epidemiological studies, clinical trials, and mechanistic research and evaluated the effectiveness and limitations of mitigation strategies, such as product reformulation, front-of-pack labeling, pricing policies, and community-based nutrition programs. Consistent evidence indicates that higher UPF intake is associated with a greater risk of micronutrient inadequacy, low-grade systemic inflammation and oxidative damage, measurable advances in biological age relative to chronological age, and higher risk of functional impairment. Although evidence linking UPF consumption with health and aging outcomes is steadily increasing, several gaps remain, including the underrepresentation of low- and middle-income settings, the lack of long-term intervention trials with aging and functional endpoints, the heterogeneous assessment of UPFs, and the limited use of mechanistic biomarkers and “omics” approaches in population studies. We suggest an interdisciplinary research approach that combines nutritional epidemiology with validated aging outcomes. In parallel, practical policy measures should prioritize minimally processed foods, fortify regulatory frameworks, and customize community and clinical interventions to meet the needs of older adults, thereby safeguarding healthy aging.

## Graphical abstract

**Figure:**
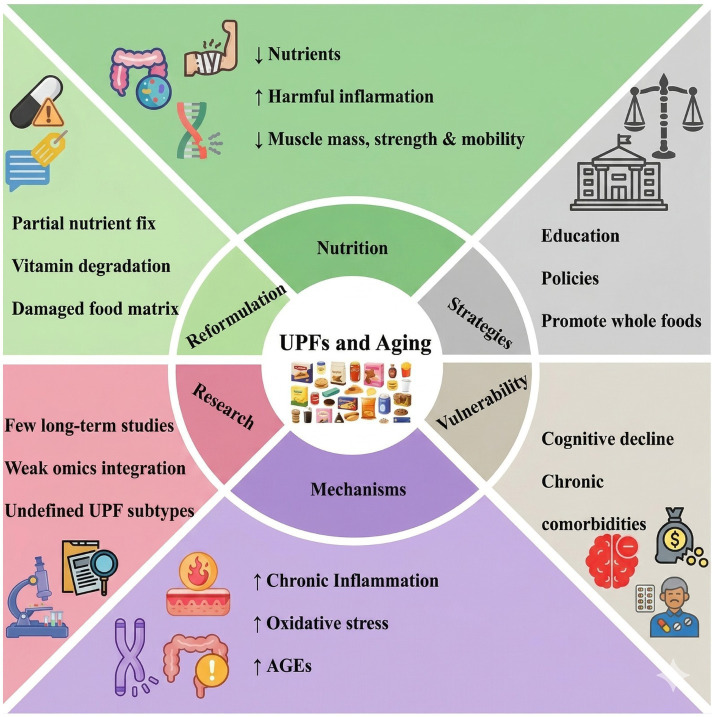

## 1 Introduction

An increasing number of large-scale studies documenting consumption trends provide strong evidence for sustained growth in UPF consumption (1–3). Over the past few years, intake has risen dramatically worldwide, posing a serious public health concern due to its significant impact on the emergence of chronic diseases and overall population health (2, 4, 5). Among high-income countries, UPF consumption is the highest in the United States (57.5% of total energy intake from UPFs) and the United Kingdom (56.8%) (6). Across Europe, more than two-fifths of total purchased dietary energy in nations such as Belgium, Germany, Finland, and Ireland is derived from UPFs, according to national household budget surveys (7). By contrast, Italy reports among the lowest levels, at approximately 10% (6). In low- and middle-income countries, although intake has traditionally been lower, UPF consumption is steadily increasing. For example, nations such as South Korea (26.1%), Colombia (15.0–15.9%), Mexico (24.9–30.0%), and Brazil show (varying from 15.0 to 29.6%) intermediate to lower levels of consumption compared with other non-Western settings (6). Notably, UPF consumption is relatively high in South Africa (40%) and Japan (30%), indicating that this dietary pattern is not exclusive to Western nations (8). A meta-analysis of nationally representative samples (7) indicates that high UPF consumption disrupts dietary balance. In particular, greater dietary shares of UPFs are associated with increased intakes of free sugars, total fats, and saturated fats, along with decreased intakes of fiber, protein, numerous vitamins and minerals, thereby compromising overall diet quality (7). Several mechanisms underlying the high consumption of UPFs have been recently highlighted. These products are typically energy-dense and contain high levels of added sugars, fats, and sodium, promoting excessive energy intake and reducing satiety signals (9). In addition, UPFs are formulated to maximize palatability with flavor enhancers, appealing textures, and aromas that stimulate hedonic eating and increase their rewarding properties (10). Experimental evidence also suggests that such sensory and compositional characteristics may activate neural reward pathways involved in motivation and food reinforcement, encouraging repeated consumption beyond actual needs (11). Moreover, the rapid digestibility and high bioavailability of nutrients in many UPFs may further strengthen preferences for these foods via reward-related mechanisms (9).

The observed transition from lower to greater UPF consumption levels across different countries suggests a potential tendency for low- and middle-income countries to follow the patterns observed in high-income countries, particularly in the absence of decisive governmental action (12). The World Health Organization’s (WHO) proactive proposal for experts to develop guidelines regulating UPF consumption further emphasizes this rising public health risk (13). Accordingly, low- and middle-income countries have a critical window of opportunity to implement preventive actions aimed at halting the widespread adoption of diets high in UPFs and mitigating significant future public health impacts and costs.

In addition, the public health implications of high UPF consumption have been reported in multiple studies. The most recent meta-analysis, which included 18 prospective cohort studies with 1,148,387 participants and 173,107 deaths, reported that individuals in the highest category of UPF intake had a 15% higher risk of all-cause mortality (HR = 1.15, 95% CI: 1.09–1.22). Moreover, each 10% increase in UPF consumption was associated with a 10% increased risk of all-cause mortality (HR = 1.10, 95% CI: 1.04–1.16). Subgroup analyses revealed that this association was stronger among male individuals than among female individuals (5). Furthermore, an umbrella review of systematic reviews and meta-analyses of observational studies reported that higher consumption of UPFs is associated with a higher risk of several chronic conditions, including type 2 diabetes, obesity, as well as adverse mental and sleep outcomes (14), highlighting the need for robust population-based and public health measures to effectively reduce UPF intake.

Several demographic differences have also been observed; younger individuals, especially teenagers, tend to consume UPFs at higher rates than older adults. Socioeconomic disparities further contribute to this pattern, as groups with lower socioeconomic status generally consume larger amounts of UPFs (5), although this association varies across countries (15). Importantly, early dietary patterns may shape long-term health trajectories, as evidenced by the consistent finding that younger adults and adolescents consume more UPFs than older populations. This pattern suggests that public health initiatives aimed at reducing UPF intake should prioritize younger individuals (16).

The sheer number of health parameters directly associated with UPF exposure, encompassing mortality, cancer, mental, respiratory, cardiovascular, gastrointestinal, and metabolic health, underscores that UPFs are not merely contributing to isolated health issues (17, 18). This suggests that UPFs fundamentally disrupt multiple physiological pathways, leading to a cascade of negative health outcomes rather than simply causing nutrient deficiencies. Therefore, public health strategies should recognize UPFs as a broad, systemic threat to health, requiring comprehensive and multi-pronged interventions rather than focusing on single nutrient targets or specific diseases. Such strategies should also account for the fact that the impact of UPFs is not uniform across all categories, emphasizing the need to distinguish between different types of UPFs based on their specific health risks, rather than advocating for universal restriction of all item classified as UPF (19).

This review aims to analyze the impact of UPFs on nutritional status, functional decline, and biological aging, while discussing possible strategies for intervention and regulation, as well as future trends in this area of research.

## 2 Why adults and older adults are particularly vulnerable

**Figure 1:**
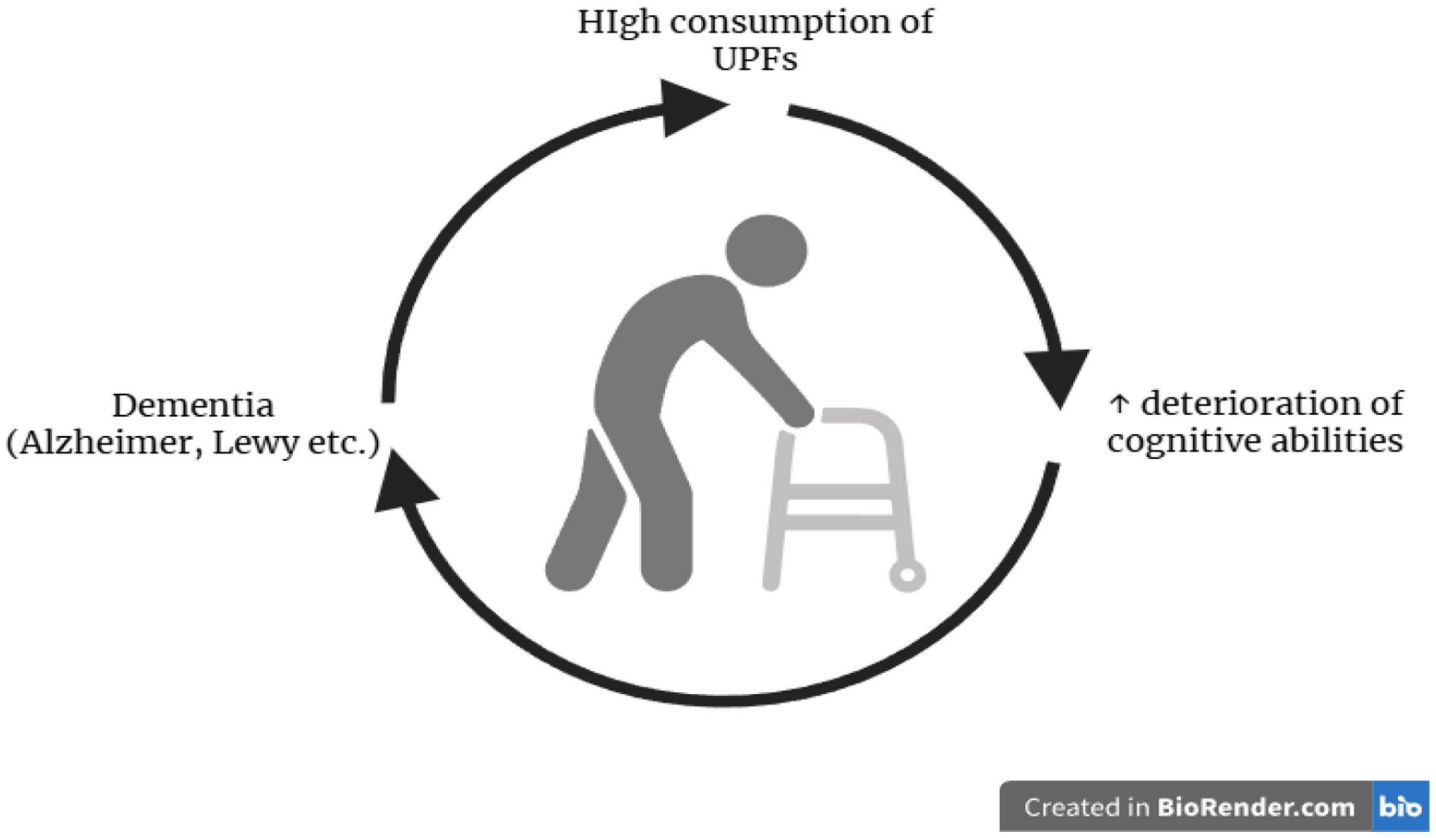
High consumption of UPFs has been linked to an increased rate of progression of cognitive decline in older adults, according to research, exacerbating the risk of developing dementia. Once dementia develops, individuals affected by the condition often become increasingly dependent on such products, as they are available in a wide range of easy-to-consume formats and contain various additives that enhance their palatability, making them a particularly attractive option. This vicious cycle is illustrated in the figure.

Adults and older adults are particularly vulnerable to the adverse effects of UPF consumption due to age-related physiological changes, the formulation properties of UPFs, and the well-documented health risks associated with these products (Figure 1) (20–22). In adulthood, chronic fatigue, whether related to aging itself or to comorbid conditions, can reduce both the capacity and motivation to prepare fresh, nutrient-dense meals. UPFs, in contrast, are widely available in ready-to-eat formulations, relatively inexpensive, and have long shelf lives, making them attractive alternatives despite their long-term health consequences (21, 23). Furthermore, chewing and dental health also influence dietary choices. The preparation of a food bolus requires sufficient jaw strength and adequate dentition, both of which may be compromised in older adults. This can often reduce the intake of meat and other firm foods, increasing the risk of macronutrient deficiencies. UPFs, which require little chewing and are easy to swallow, may become a convenient option for older individuals and caregivers alike, further reinforcing their consumption (20, 24).

Cognitive decline represents an additional vulnerability. Whether it arises early due to underlying conditions or later as part of the natural process, cognitive impairment can impair food choices and dietary habits. High UPF consumption has been associated with chronic inflammation and oxidative stress, which exacerbates mild cognitive impairment, dementia, and mood disorders such as depression and anxiety (24). A recent study reported that high UPF consumers had up to a 10% higher risk of developing depressive symptoms compared with controls (25). Longitudinal evidence further supports these associations, suggesting a vicious cycle in which cognitive decline increases reliance on UPFs, while the additive and reward-stimulating properties of these foods further perpetuate dependency (26).

Sociodemographic and environmental factors may also influence the UPF consumption patterns in the older population. Urban environments tend to promote greater UPF availability and cultural acceptability (27). A Brazilian study identified education level and urban residence as predictors of UPF intake, likely reflecting reduced access to healthy options and increased reliance on inexpensive processed plant-based foods such as corn chips, cookies, and fast food (28). Conversely, some studies report a counterintuitive association between higher education and higher UPF consumption. This may be explained by time constraints, greater purchasing power, convenience, and higher exposure to intense marketing campaigns from transnational food companies (27, 28).

Interestingly, research in Colombia highlights how urban residents are more exposed to food marketing and more inclined to adopt novel, often artificial flavors, while rural populations maintain traditional culinary practices and greater reliance on natural, minimally processed foods due to cultural continuity and affordability (29).

Taken together, these physiological, cognitive, and social determinants reveal why adults and older adults are disproportionately susceptible to UPF consumption. Their vulnerabilities stem not only from biological aging and disease but also from structural and environmental contexts that favor the accessibility, affordability, and acceptability of UPFs.

## 3 Nutritional and functional consequences of UPF consumption

High consumption of UPF is strongly associated with a lower intake of essential nutrients. Multiple studies have reported an inverse association between UPF consumption and dietary protein, fiber, and vitamins (A, C, D, E, B6, B12, riboflavin, niacin, and thiamin), as well as minerals such as calcium, magnesium, zinc, and iron intake (7, 30, 31). For example, a Brazilian study found that individuals with high UPF consumption had more than twice the risk of vitamin D deficiency compared to controls; minimally processed foods provided tenfold higher vitamin D content than UPFs (3.43 vs. 0.34 μg) (32). Similarly, in the SUN cohort, individuals with the highest UPF intake (>3 servings/day) consumed less protein, fiber, potassium, calcium, magnesium, and olive oil, as well as fewer fruits and vegetables, compared with those in the lowest intake group (24). Comparable findings from Kenya showed significantly lower calcium intake in individuals with high UPF consumption (282.2 vs. 344.7 mg/1000 kcal) (33).

In Spain, magnesium intake was markedly lower among high UPF consumers (408.3 ± 135.3 vs. 488.5 ± 142.4 mg/day, *p* < 0.001) (24). Other studies confirm consistent reductions in calcium, magnesium, and zinc intake, alongside deficits in fiber and protein (2, 33–35). At the same time, UPF-rich diets are characterized by higher intakes of saturated fatty acids (SFAs). One study found that individuals in the highest quartile of UPF intake had significantly higher percentages of SFA (12.7% vs. 10.4%) compared with those in the lowest quartile (24).

**Figure 2:**
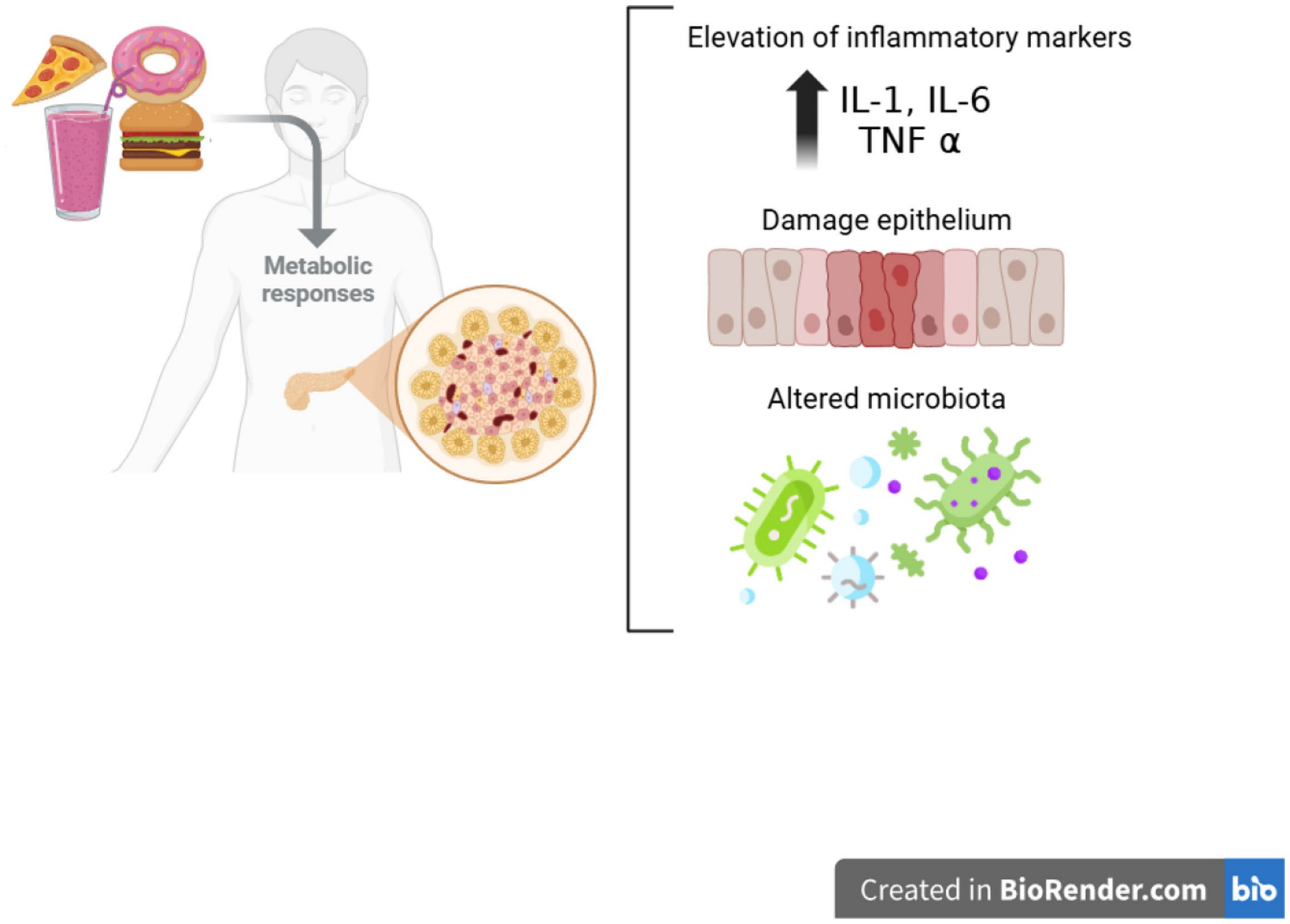
Ultra-processed food consumption triggers adverse metabolic responses. These responses lead to three main consequences: (1) Elevation of systemic inflammatory markers (IL-1, IL-6, and TNF-*α*), which establishes a persistent inflammatory state; (2) damage to the intestinal epithelium, evidenced by the alteration and loss of integrity of epithelial cells; and (3) modification of the composition and balance of the intestinal microbiota, affecting gastrointestinal homeostasis.

These nutrient imbalances contribute to major functional consequences. As summarized in Figure 2, the three principal consequences of UPF consumption comprise (1) elevated levels of pro-inflammatory markers (IL-1, IL-6, and TNF-*α*), (2) epithelial barrier damage, and (3) alterations in the gut microbiota. In particular, deficiencies in antioxidant vitamins (C, D, and E) are linked to elevated inflammatory markers, including C-reactive protein and interleukin-6 (36, 37). The gut microbiome is a central mechanism regulated by dietary factors, including UPFs (38). UPF consumption alters microbiota composition, reduces epithelial barrier integrity, and increases immune activation. Lower fiber intake, characteristic of dietary patterns high in UPFs, is consistently associated with gut disruption (38, 39) and impaired production of short-chain fatty acids (SCFAs), thereby compromising gut and immune homeostasis (40). Beneficial species such as *Bifidobacterium* spp. and *F. prausnitzii* are depleted, promoting chronic inflammation, impaired nutrient absorption, and epigenetic modifications within the intestinal wall (20, 41, 42).

Furthermore, low-grade systemic inflammation, characterized by persistent subclinical immune activation, has emerged as a hallmark of high UPF consumption. This state promotes oxidative stress, aberrant metabolic activity, and anti-apoptotic signaling, creating a pro-tumorigenic environment (43, 44). Additionally, certain UPF components, such as artificial sweeteners including aspartame and sucralose, have been associated with insulin resistance and endocrine disruption (38, 43–47).

Higher UPF shares in the diet have been linked to several functional consequences. In particular, a recent systematic review with meta-analysis investigated the relationship between UPF consumption and age-related muscle outcomes, including frailty, sarcopenia, low muscle mass, and low muscle strength; using data from 29 studies, the meta-analysis found that higher UPF intake was significantly associated with an increased risk of frailty in cohort studies (RR = 1.40, 95% CI 1.25–1.58), whereas cross-sectional studies showed an association with low muscle strength but not with frailty, sarcopenia, or low muscle mass. Dose–response analysis indicated that each 100 g/day increase in UPF intake was linked to a 3% higher risk of frailty, suggesting that the regular consumption of ultra-processed foods may negatively affect muscle health and functional independence in older adults (48). Another systematic review with meta-analysis synthesizing evidence from 43 studies found that higher UPF consumption was consistently associated with increased frailty risk, multimorbidity, systemic inflammation, and sarcopenia, with dose-dependent associations showing reductions in grip strength, slower gait speed, lower muscle mass indices and elevated inflammatory markers. Overall, these findings suggest that UPF intake represents a modifiable risk factor for frailty and muscle deterioration, highlighting the reduction of UPF consumption as a potential strategy to improve aging-related health outcomes (49). Regarding accelerated aging, two large population-based studies, one in the US and one in Italy, have shown that increased biological age, as determined by validated biomarker-based algorithms such as PhenoAge (deep neural network models using circulating biomarkers), is independently correlated with higher UPF consumption (50, 51). Even after controlling for conventional dietary quality indices, individuals in the top quintile of UPF intake in the Moli-sani study had a mean biological age that was 0.34 years higher than those in the lowest quintile (50). According to NHANES data, individuals in the highest quintile of UPF consumption are more than 1 year older biologically than people in the lowest quintile, and every 10% increase in energy from UPFs is associated with a 0.21-year increase in biological age (51). Interestingly, adjusting for overall diet quality only partially reduces these relationships, indicating that biological aging may be influenced by elements inherent in food processing that go beyond nutritional content. Finally, sarcopenia has also been linked to UPF consumption in older adults, likely due to the pro-inflammatory properties and low nutrient density of these foods (15, 16, 51), demonstrating that high UPF consumption is associated with increased risk of micronutrient inadequacy and is independently linked to accelerated biological aging (51). Moreover, higher UPF intake (>3 servings/day) was associated with a twofold increased odds of shorter telomeres among older adults in the SUN cohort, and NHANES analyses confirmed a significant association between UPF consumption and accelerated phenotypic aging (PhenoAgeAccel) (24, 52). These findings suggest that UPFs contribute to both nutritional inadequacies and functional decline.

## 4 How to improve the nutritional adequacy of UPFs?

Improving the nutritional quality of UPFs has become a key objective in nutrition science and public health due to the growing evidence linking the consumption of these products with adverse health outcomes. Product reformulation is widely proposed as a strategy to mitigate the negative nutritional profile of UPFs while maintaining their sensory properties. Several approaches could be undertaken, including modifying ingredient composition, reducing harmful nutrients, and incorporating more beneficial components (77–81).

One of the most common reformulation strategies involves reducing sodium, added sugars, and saturated fats. Many processed food categories, including breads, snacks, and ready meals, have undergone reformulation aimed at lowering these components without substantially altering taste or consumer acceptance. Population-level modelling studies suggest that gradual sodium and sugar reductions in processed foods could significantly improve dietary patterns and reduce the risk of chronic diseases such as hypertension and cardiovascular disease (53). However, some authors note that nutrient-focused reformulation may not fully address the broader health implications associated with the degree of food processing (54). Another strategy involves the substitution of artificial additives with natural ingredients. Recent research has explored the use of plant-derived compounds, fermentation products, and natural antioxidants as alternatives to artificial preservatives, colorants, and flavor enhancers.

Reformulation can also involve replacing highly refined ingredients with minimally processed alternatives. The incorporation of whole grains, legumes, and vegetable-based ingredients instead of refined starches or sugars can enhance fiber content and improve overall nutrient density (54). Overall, these reformulation strategies demonstrate how technological innovation and ingredient substitution could contribute to improving the nutritional profile of ultra-processed foods, although their effectiveness would ultimately depend on the extent and nature of the modifications applied. Given the rising global consumption of UPFs, policymakers should advocate for nutritional reformulation of these products by the food industry in order to improve their nutritional profile.

## 5 Strategies for intervention and policy response

UPFs are consistently associated with adverse nutritional and health outcomes, underscoring the need for comprehensive strategies that act simultaneously at the community, clinical, and policy levels. Effective responses should promote minimally processed foods, empower individuals with knowledge and skills, and strengthen regulatory frameworks that align food environments with public health objectives.

### 5.1 Community-level strategies

Evidence from community-based and primary care interventions highlights the importance of reshaping food environments and building nutrition literacy. A randomized 12-month community trial showed that participants in the highest quartile of UPF consumption gained significantly more weight (≈0.36–0.47 kg) than those in the lowest quartile, regardless of intervention group, reinforcing the need to reduce energy intake from UPFs (55). Community interventions that combine education, cooking skills, access to fresh foods, and participatory evaluation have demonstrated promising results. Sustainable impact, however, requires long-term follow-up (≥12 months) with outcome monitoring that includes dietary intake, anthropometry, and quality of life (56, 57).

### 5.2 Clinical-level strategies

Controlled reduction of UPF intake has been associated with improvements in body weight, energy balance, and metabolic health, suggesting a causal effect of the qualitative dietary reform beyond calorie counting (56). Clinical practice should encourage the substitution of UPF-based meals with minimally processed alternatives and the implementation of a personalized dietary prescription, supported by regular monitoring of anthropometric and biochemical indicators every 4–12 weeks (55, 58). While short-term benefits for weight and metabolic outcomes have been observed, longer-term trials are needed to determine sustained effectiveness (55, 56).

### 5.3 Policy and regulatory strategies

The escalating global consumption of UPFs underscores the need for stronger public health interventions aimed at improving the overall quality of diets. In this context, policymakers should actively promote strategies that encourage the food industry to reformulate these products in order to enhance their nutritional composition. Such reformulation initiatives should prioritize the reduction of detrimental ingredients, including the excessive levels of added sugars, sodium, and unhealthy fats, while simultaneously increasing the presence of beneficial nutrients. By improving the nutritional profile of UPFs, these measures may help mitigate the adverse health effects commonly associated with their widespread intake.

Furthermore, an analysis of the regulatory activity in the United States (US) indicates that few policies explicitly target UFPs. The majority of these policies merely describe them as inconsistent with healthy eating recommendations, and very few provide an operational definition. These regulatory gaps highlight the urgent need for clearer policy measures, including front-of-package warning labels on critical nutrients regardless of fortification claims, and strict marketing restrictions on foods with unfavorable nutritional profiles (59).

In summary, interventions to reduce UPF consumption must operate simultaneously at the community, clinical, and policy levels. Best practice involves nutritional reformulation of UPFs and redirecting them toward minimally processed foods, while integrating educational, clinical, and regulatory strategies to promote sustainable dietary improvements, reduce barriers, and enhance healthier aging trajectories (60, 61). Therefore, coordinated efforts between policymakers and the food industry are essential to foster healthier food environments and support improved population health outcomes.

## 6 Research gaps and future directions

While observational studies suggest an association between UPF intake and adverse aging outcomes, causal inference remains limited by the lack of prospective designs with functional endpoints (Table 1) (48, 62). A 2025 meta-analysis found that high UPF intake was associated with a 40% increase in the odds of frailty and a 32% increased risk of low muscle strength. However, no consistent association was observed for conditions related to aging, such as sarcopenia, in this study (48). Stronger associations were found in cohort studies compared to cross-sectional designs or randomized trials, indicating a possible correlation with the study design (48). These findings highlight the need for longitudinal and interventional studies that incorporate aging-related outcomes measured with validated tools (63, 64). The relatively short follow-up periods and the possible presence of confounders further restrict causal interpretation.

**Table 1:** Gap categories and future direction research in UPFs.

Gap category	Description	Proposed research direction
Longitudinal evidence	Lack of long-term studies evaluating UPFs and functional or cognitive decline	Establish prospective cohorts with repeated exposure and validated aging endpoints
Causal inference	Short RCT durations and absence of temporality limit causal interpretation	Design trials with extended follow-up and functional or cognitive outcomes
Mechanistic pathways	Biomarkers of aging (e.g., IL-6, telomeres, and epigenetic clocks) are rarely measured	Include mechanistic biomarkers in cohort and intervention studies
Geographic representation	Underrepresentation of LMICs and need for context-adapted UPF classification	Conduct UPF-aging studies in diverse settings using culturally relevant tools
Population-specific barriers	Few interventions target older adults who face barriers to dietary change	Design interventions tailored to the physical, cognitive, and economic context of aging
UPF subtype differentiation	UPFs are treated as a homogeneous group despite nutritional variation	Conduct subtype analyses to distinguish harmful vs. reformulable UPFs

UPF, ultra-processed food; RCT, randomized controlled trial; LMIC, low and middle-income countries.

Although recent trials such as UPDATE and ANCOVIA demonstrated adverse metabolic and vascular effects after short-term UPF consumption, they did not assess long-term changes in frailty, disability, or cognitive function (33, 64).

Mechanistic pathways remain underexplored. Although inflammation, oxidative stress, endocrine disruption, mitochondrial dysfunction, and gut dysbiosis have been proposed, a limited number of studies have assessed relevant biomarkers such as IL-6, TNF-*α*, C-reactive protein, telomere length, or DNA methylation (65, 66). Gut microbiota has emerged as a central player in mediating the inflammatory and metabolic effects of diet; however, few UPF-aging studies incorporate microbial or metabolomic profiling (67). Routine aging biomarkers, such as inflammatory markers or grip strength, are feasible to implement in nutritional studies but are rarely included. Promising tools such as epigenetic clocks, mitochondrial assays, and cellular senescence panels could provide additional insights into biological aging but remain underutilized in UPF-focused research (66). Their integration into future studies would help elucidate whether UPFs accelerate physiological decline via molecular aging pathways.

Furthermore, geographic representation in UPF-aging research is limited. The majority of evidence originates from high-income countries, despite rising UPF intake in low- and middle-income settings. Recent data from China and South Korea demonstrate the feasibility of large-scale cohort studies in diverse populations (20, 62, 68). However, the transferability of existing UPF classification systems, particularly NOVA, requires refinement to reflect local food systems. Culturally adapted and validated tools for UPF exposure assessment are critical to ensure comparability and policy relevance (6, 69, 70).

It should also be noted that older adults face distinct structural and behavioral barriers to reducing UPF intake, including mobility limitations, cognitive decline, and fixed incomes (27). However, few dietary interventions have been designed for or tested in this population. Future programs must consider age-specific challenges and support systems, such as assistance with food preparation, targeted education, and improved food access.

Finally, UPFs are frequently treated as a homogeneous group despite significant variability in their composition, degree of processing, and nutrient profiles. This oversimplification may obscure important differences in health effects. Subtype analyses are needed to identify which classes of UPFs, such as plant-based meat analogues, fortified cereals, or energy-dense snack foods, are most strongly associated with adverse outcomes (51, 58). Such data could guide reformulation efforts and support more precise dietary recommendations.

Addressing these gaps will require interdisciplinary, globally inclusive research that combines robust dietary assessment with validated functional and biological aging outcomes. This evidence is urgently needed to inform public health guidance and food policy for aging populations.

## 7 Discussion

This narrative review highlights the growing evidence linking UPF consumption to nutritional inadequacies, functional decline, and accelerated aging, including frailty, reduced muscle strength, and cognitive decline. These conclusions are further supported by systematic reviews demonstrating that higher UPF consumption is linked to a higher risk of dyslipidemia, abdominal obesity, frailty, and poorer cognitive function in adults over 60 (48, 71). While healthier dietary patterns, such as the Mediterranean diet, are linked to reduced risk of some cancer types, slowed diabetes progression, lower frailty, and improved muscle strength, high shares of UPF in the diet appear to exert the opposite effect (64, 72–74). Interestingly, a recent multinational survey conducted in Mediterranean and nearby countries showed that adherence to the Mediterranean diet has decreased, while consumption of ultra-processed foods has increased, reflecting a similar trend worldwide (75).

The detrimental impact of UPFs is attributed to multiple contributing factors. Their substitution of nutrient-dense, minimally processed foods leads to lower intake of essential proteins, fiber, and micronutrients critical for maintaining muscle mass, metabolic health, and cognitive resilience. Moreover, UPF-rich diets increase exposure to additives, advanced glycation end-products, and excess sodium and sugar, all of which are implicated in aging-related biological pathways (48).

Although several methodological limitations make these findings difficult to interpret. Many studies used dietary recall tools that were not specifically designed to classify UPFs, raising the risk of misclassification (76). In addition, UPF intake has often been expressed as a percentage of total energy, which can obscure real consumption levels; reporting both the percentage of energy and the grams per day could give a clearer picture (58). Finally, overlapping cohorts and the possibility of residual confounding, despite adjustments for factors such as physical activity and chronic illness, remain important concerns (71). Other limitations include limited subgroup analyses by UPF subtype or sociodemographic strata (71).

Short-duration trials have generally produced null or inconsistent results, likely due to insufficient follow-up, small or homogeneous samples, and lack of functional aging endpoints (20, 64). While plausible biological mechanisms—including chronic inflammation, oxidative stress, mitochondrial dysfunction, and endocrine disruption—have been proposed, these remain under investigation in aging-focused nutrition research. There is a particular need for biomarker-based studies and dietary intervention trials targeting these pathways in older adults (20, 48).

### 7.1 Strengths and limitations of the current evidence

The emerging literature examining UPF intake in relation to aging outcomes benefits from a growing number of large-scale prospective cohorts with standardized dietary assessment methods and validated health outcomes (48, 71). These studies provide valuable population-level insights and enable the adjustment for multiple confounders. However, the evidence base remains constrained by notable limitations. Many studies are observational, making causal inference challenging, and are geographically concentrated in high-income countries, limiting generalizability. Short-term randomized trials, while informative for acute metabolic effects, are often underpowered for detecting changes in functional aging endpoints (20, 64).

The heterogeneity in UPF exposure definitions, dietary assessment tools, and outcome measures complicates cross-study comparisons. Few studies account for UPF subtypes, which may differ substantially in nutrient composition and health effects, or for behavioral factors such as sedentary time that cluster with high UPF intake (71). Addressing these limitations will require harmonized measurement protocols, more diverse study populations, and the integration of biomarker-based and mechanistic endpoints into long-term interventions.

### 7.2 Overall interpretation and implications

**Figure 3:**
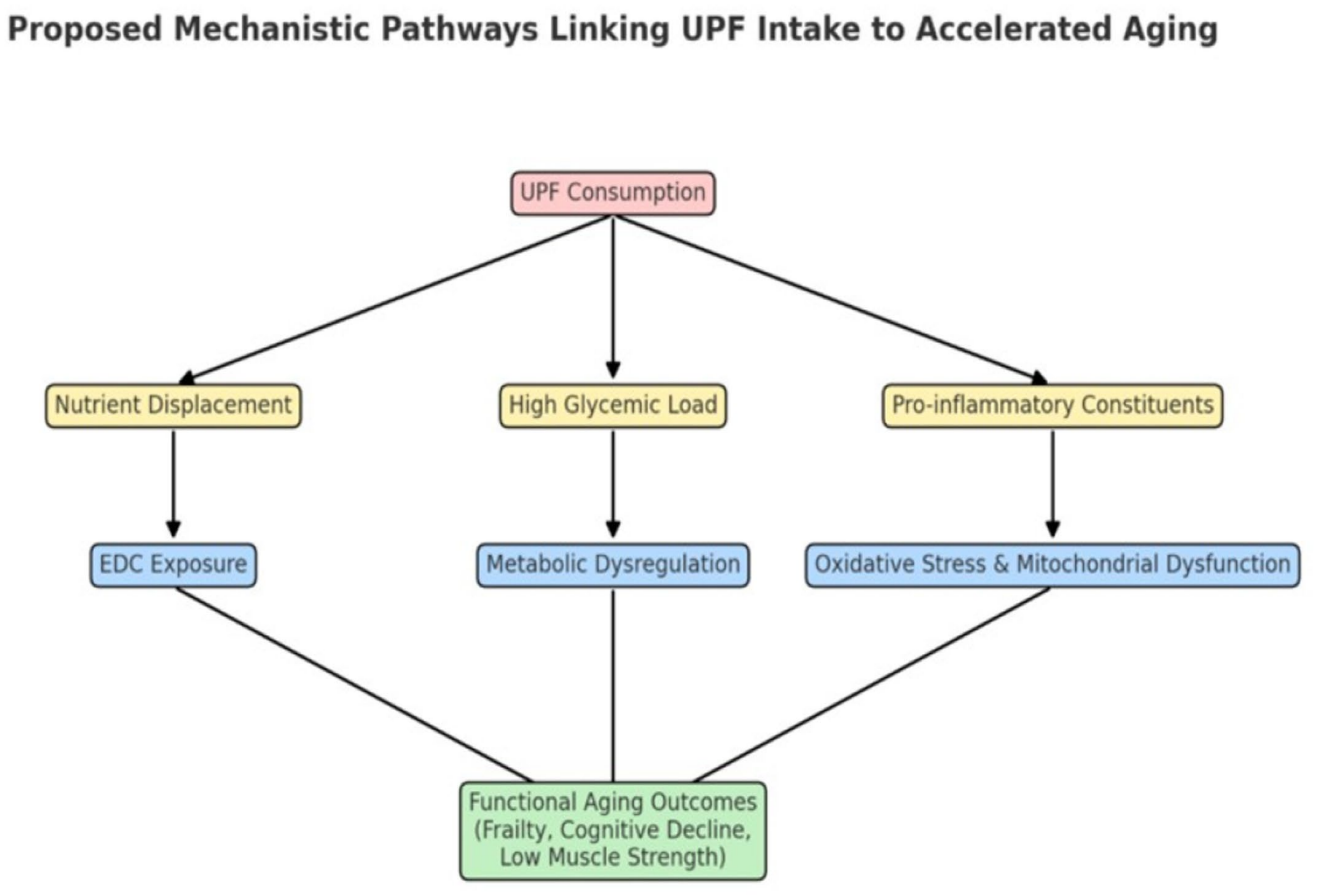
Conceptual framework linking ultra-processed food (UPF) consumption to adverse aging outcomes. UPF intake contributes to nutrient displacement, high glycemic load, and pro-inflammatory constituents, as well as exposure to endocrine-disrupting chemicals (EDCs). These mechanisms promote metabolic dysregulation, oxidative stress, and mitochondrial dysfunction, ultimately leading to functional aging outcomes, including frailty, cognitive decline, and reduced muscle strength.

Taken together, the available evidence supports a consistent association between high UPF consumption and multiple indicators of accelerated aging, including frailty, cognitive decline, and reduced physical function. While causality cannot yet be established, the convergence of epidemiological and mechanistic data underscores the potential public health importance of limiting UPF intake in older populations. Advancing this field will require interdisciplinary approaches that combine robust dietary assessment, longitudinal follow-up, and functional and biological aging endpoints to clarify both the magnitude and mechanisms of risk (see Figure 3; Table 2).

**Table 2:** Summarizes priority research, clinical, and policy actions to reduce UPF exposure, protect vulnerable populations, and promote healthy aging.

Domain	Recommendation	Stakeholders
Research Evidence	Prioritize longitudinal cohorts and extended RCTs that use validated aging outcomes (frailty, cognition, and biomarkers) and rigorously adjust for confounding	Researchers and funding agencies
Classification and Labeling	Standardize and refine UPF classification and labeling to clearly distinguish harmful subtypes	Policy makers and regulators
Clinical and Community Practice	Embed UPF reduction into geriatric care and community nutrition programs, tailored to socioeconomic and cultural contexts	Clinicians, dietitians, and non-governmental organizations
Reformulation strategies	Safeguard reformulation strategies to improve the nutritional quality of UPFs	Policy makers and global health agencies
Integrated Strategies	Implement coordinated fiscal, educational, and regulatory policies to curb UPF exposure and promote minimally processed foods	Governments and health systems

### 7.3 Policy and practice implications

Together, these recommendations provide a roadmap for translating current evidence on UPFs into effective public health action to support healthy aging.

## 8 Key conclusions

According to the reviewed literature, a high intake of UPFs is consistently associated with oxidative stress, systemic inflammation, functional decline, and micronutrient deficiencies, all of which hasten biological and phenotypic aging. Effective strategies aiming at reformulating the nutritional quality of UPFs are warranted to improve UPFs in terms of the overall diet quality and health outcomes. To address socioeconomic determinants of nutrition, particularly for adults and older people, public health responses should prioritize the promotion of minimally processed foods, strengthen regulatory frameworks, and develop culturally relevant interventions. Beyond cross-sectional evidence, future research should incorporate longitudinal and intervention studies with validated biomarkers of aging and functional outcomes. Reducing the demographic burden of UPFs and promoting healthy aging requires a comprehensive, multidisciplinary approach.
